# Relationship between Step Characteristics and Race Performance during 5000-m Race

**DOI:** 10.3390/sports9090131

**Published:** 2021-09-17

**Authors:** Hiromasa Ueno, Sho Nakazawa, Yohsuke Takeuchi, Masaaki Sugita

**Affiliations:** 1 Graduate School of Health and Sport Science, Nippon Sport Science University, 7-1-1 Fukasawa, Tokyo 158-8508, Japan; icc05779@nifty.com; 2 Faculty of Sport Science, Nippon Sport Science University, 7-1-1 Fukasawa, Tokyo 158-8508, Japan; s-nakazawa@nittai.ac.jp (S.N.); m-sugita@nittai.ac.jp (M.S.)

**Keywords:** step length, step frequency, contact time, fatigue, running economy

## Abstract

This study examined the relationship between step characteristics and race time in a 5000-m race. Twenty-one male Japanese endurance runners performed a 5000-m race. Step length, step frequency, contact time, and flight time of two gait cycles (i.e., four consecutive ground contacts) were measured every 400-m by using high-speed video image. Moreover, step length was normalized to body height to minimize the effect of body size. In addition to step characteristics on each lap, the averages of all laps and the per cent change from the first half to the second half were calculated. The average step frequency and step length normalized to body height correlated significantly with the 5000-m race time (*r* = −0.611, *r* = −0.575, respectively, *p* < 0.05 for both). Per cent changes in contact time and step length correlated significantly with the 5000-m race time (*r* = 0.514, *r* = −0.486, respectively, *p* < 0.05 for both). These findings suggest that, in addition to higher step frequency and step length normalized to body height, smaller changes in step length during a given race may be an important step characteristic to achieving superior race performance in endurance runners.

## 1. Introduction

Running economy, which is a key determinant of running performance, is strongly related to biomechanical factors, including kinematic, kinetic, mechanical, and morphological properties of skeletal muscles, tendons, and joints [1,2,3,4,5,6,7]. Tartaruga et al. [4] performed a multivariate analysis using several biomechanical characteristics (i.e., number of steps, kinematic, kinetic, and electromyographic variables) and determined that higher step frequency (SF) and shorter step length (SL) were associated with better running economy. Therefore, running with a higher SF and shorter SL is considered an important running technique for achieving higher running performance. However, although Folland et al. [2] determined a similar relationship of running economy with SF and SL, they showed that SF and SL did not correlate with the personal best time in endurance runners. Typically, the running economy is measured during submaximal running at a speed below the ventilatory–lactate threshold [1]. Moreover, step characteristics, including SF and SL, change depending on the running speed [8,9,10]. Taken together, it is necessary to analyze step characteristics while running at a speed near the race pace to clarify the relationship of SF and SL with running performance.

Anthropometric characteristics (i.e., leg length) are particularly relevant for determination of SL [11,12] and are found to be race-dependent [13,14]. For example, previous studies report that runners of North African descent run with a longer SL than those of Asian or European descent, probably because of a longer average leg length [13,14]. Running performance has been previously associated with leg length; runners with a greater leg length are able to increase SL with less associated energy consumption [15,16]. Therefore, the effect of step characteristics on running performance may vary according to the intrinsic morphology of the endurance runner, and this should be taken into consideration when developing a training program.

Fatigue influences step characteristics because of the inability to maintain a good level of force production [17,18,19]. The running speed is determined by multiplying SL by SF. Thus, changes in step characteristics due to running-induced fatigue during the race may affect the running speed and alter performance. While performing to achieve a higher running speed, increasing SL required greater work of the limb joints than increasing SF [20]. In addition, the level of force production from the limb muscles decreased with fatigue development during running [17,18,21]. Previous studies have shown that SL decreased at the end of the race compared to the start of the race; however, SF did not change in 5000-m and 10,000-m races [18,22]. In turn, a fatigue-related decrease in SL may reduce running speed and result in poor race performance. Considering these previous findings, SL, rather than SF, may be more prone to variations with fatigue in the race because maintaining a high SL may require a higher level of force production from the limb muscles.

This study aimed to analyze the step characteristics of Japanese endurance runners in a 5000-m race and examine the consequent relationships between step characteristics and race times. We hypothesized that higher SF is related to better running economy and better performance, as suggested by other previous studies [2,4,23]. Additionally, we hypothesized that maintaining a higher SL at a later stage of the race may be related to better race performance because SL would decrease with an increase in running-induced fatigue. Taken together, a greater knowledge of performance-related changes to step characteristics would be advantageous for developing effective training programs and understanding differences in running performance outcomes for endurance runners

## 2. Materials and Methods

### 2.1. Participants

Sample sizes used in previous studies [21] were found to be sufficient and adapted to suit the present study. Determination of a correlation between step characteristics and performance required a minimum of 11 participants, as calculated from an effect size of 0.70 (correlation between contact time and running speed [21]), α–level of 0.05, and β–level of 0.2 (80% power).

Twenty-one Japanese male endurance runners (age: 19.9 ± 1.1 years, body height: 171.2 ± 5.2 cm, body mass: 57.3 ± 4.9 kg) participated in this study. They were all well-trained and involved in regular training and competition. They were free of musculoskeletal injury, with no self-reported neurological and cardiopulmonary impairments. The subjects were informed of the experimental procedures, and they provided written consent to participate in the study. All procedures were approved by the Ethics Committee of Nippon Sport Science University (019–H193).

### 2.2. Data Collection

This study was conducted at the 273rd Nippon Sport Science University Competition (6 October 2019) and the 274th Nippon Sport Science University Competition (17 November 2019), which are given official recognition by the Japan Association of Athletics Federations. These competitions simulate a time trial format, with the aim of achieving personal records rather than pursue rankings. The participants were divided into groups according to their running levels and were instructed to attempt their personal best 5000-m running times. Each participant competed only in a 5000-m race. A high-speed video camera (DMC-FZ300, Panasonic Corp., Osaka, Japan) was placed inside the home straight to video record the runners from 360.0 to 372.5 m points. The camera was fixed using a tripod. The filming rate was set at 120 Hz, as used previously for analysis of step characteristics in long- and middle-distance races [24,25]. For calibrating the aspect ratio for vertical and horizontal measurements, a 3 m pole was used.

### 2.3. Data Analysis

Two identifiable gait cycles (i.e., four consecutive ground contacts) were analyzed on each lap from the second lap to the thirteenth lap (i.e., the final lap). Steps on the first lap were not analyzed because ground contacts could not be identified completely due to the overlapping of runners in the videos. The contact time (CT) and flight time (FT) were measured and calculated from the filming rate (1 frame = 0.0083 s). The CT and FT of two gait cycles on each lap were averaged. The mean coefficient of variation ± standard deviation (SD) of CT and FT for each lap were 2.6 ± 0.7% and 3.4 ± 1.0%, respectively. These coefficients are lower than the coefficients of variation reported in a previous study calculated using a similar methodology [26]. SF was calculated as 1/(CT + FT) in Hz. SL was measured as the length between the heel on the first ground contact to the heel on the next ground contact by using two-dimensional (2D)-four points real length conversion method which is used in the real track race situations [27,28]. For calibrating each lane, the 2D-four points calibration volume was set as 12.5 m in the running direction. The SL of two gait cycles on each lap was averaged. The mean coefficient of variation ± SD of SL for each lap was 1.7 ± 0.4%, and SL was normalized to body height (BH) to minimize the effect of body size [2,29]. Moreover, the instantaneous running speed (m/sec) was calculated by multiplying SF by SL. In addition to step characteristics being analyzed on a lap-by-lap basis, averages of the step characteristics from the second to the twelfth laps were calculated for the first half (second to the seventh lap), the second half (eighth to the twelfth lap) and all laps (second to the twelfth lap). Step characteristics in the final lap (thirteenth lap) were used independently for statistical analysis of the final sprint. Additionally, the per cent change from the first half to the second half was calculated. The step characteristics were analyzed using an image analysis software (Frame-DIAS V, DKH, Tokyo, Japan).

### 2.4. Statistical Analysis

All data are expressed as mean ± SD. The effect of time was determined by a single factor analysis of variance (ANOVA) for repeated measures across each lap. If the sphericity assumption was not met, the Greenhouse–Geisser corrections were used. Specific differences were identified using a Tukey HSD post hoc test. The η^2^ effect size was calculated to determine the magnitude of the difference in each ANOVA test. The strength of the effect sizes was interpreted as weak (η^2^ > 0.10), moderate (η^2^ > 0.25), and strong (η^2^ > 0.40) [30]. Relationships between the step characteristics and 5000-m race time were assessed using Pearson’s product moment correlation coefficient. The strength of the relationship between any two variables was interpreted as trivial (<0.10), small (0.10–0.29), medium (0.30–0.49), and large (>0.50) [30]. Statistical significance was set at *p* < 0.05. All statistical analyses were conducted using IBM SPSS software (version 19.0; International Business Machines Corp., Armonk, NY, USA).

## 3. Results

**Figure 1:**
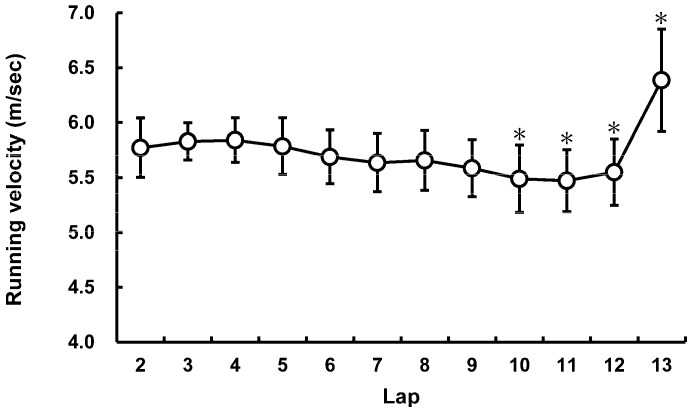
Running speed at each lap during the 5000-m race; * *p* < 0.05 significantly different from the second lap.

The mean 5000-m race time was 14 min 52 s ± 27 s (13 min 54 s to 15 min 49 s). The average running speed was 5.66 ± 0.20 m/sec (5.24 m/s to 6.12 m/s). The change in running speed is shown in Figure 1 (*F* = 28.394, *p* < 0.001, and η^2^ = 0.587).

**Figure 2:**
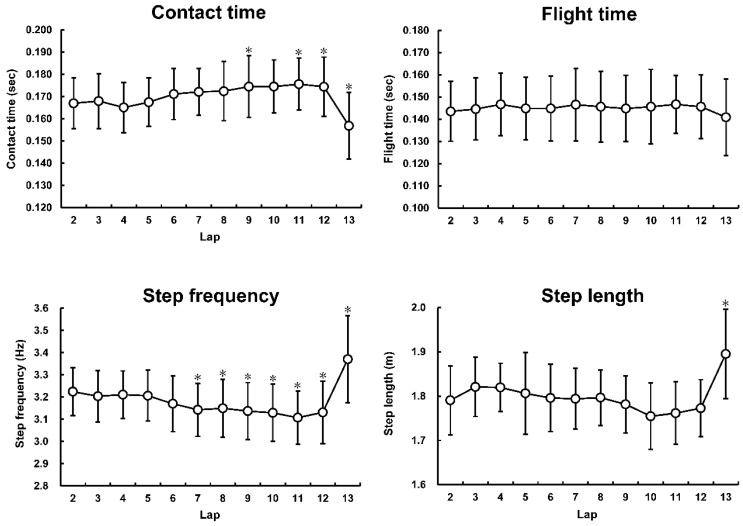
Step characteristics at each lap during the 5000-m race; * *p* < 0.05 significantly different from the second lap.

**Figure 3:**
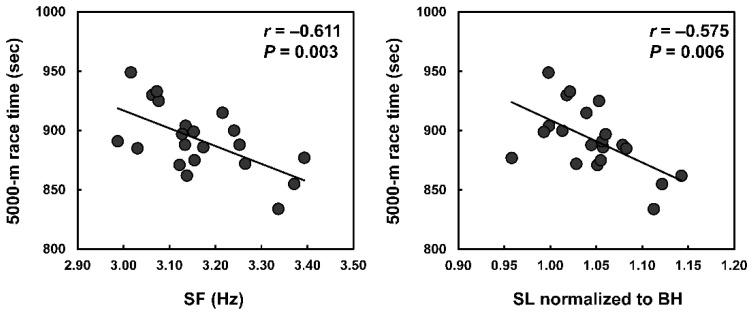
The relationship between average SF and SL normalized to BH of all laps with 5000-m race time. The strengths of the relationship were large for CT and SL normalized to BH.

The data of the step characteristics are shown in Table 1. The changes in step characteristics are summarized in Figure 2 (*F* = 12.170, *p* < 0.001, and η^2^ = 0.378 for CT; *F* = 1.311, *p* = 0.220, and η^2^ = 0.062 for FT; *F* = 21.685, *p* < 0.001, and η^2^ = 0.520 for SF; *F* = 10.443, *p* < 0.001, and η^2^ = 0.343 for SL). The average SF and SL normalized to BH were significantly correlated with the 5000-m race times (Figure 3).

**Figure 4:**
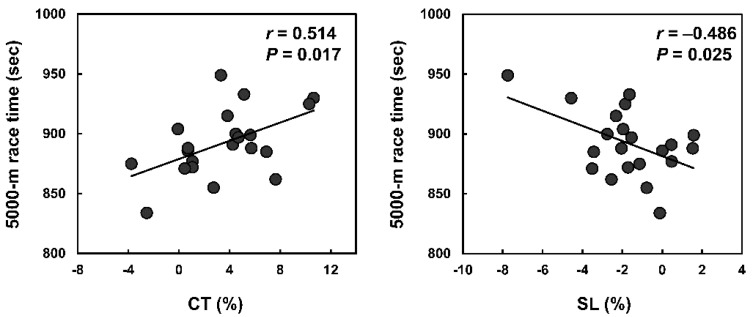
The relationships of per cent changes in CT and SL with 5000-m race time. The strengths of the relationship were large for CT and medium for SL.

In the first half of the 5000-m race, only the average SF and SL normalized to BH correlated with the 5000-m race times (*r* = −0.579 (large), *p* = 0.006; *r* = −0.488 (medium), *p* = 0.025; respectively). However, in the second half, average CT, SF, SL, and SL normalized BH correlated significantly with the 5000-m race time (*r* = 0.506 (large), *p* = 0.019 for CT; *r* = −0.615 (large), *p* = 0.003 for SF; *r* = −0.553 (large), *P* = 0.009 for SL; *r* = −0.684 (large), *p* < 0.001 for SL normalized to BH). In the final lap, SF and SL normalized to BH correlated with the 5000-m race times (*r* = −0.541 (large), *p* = 0.011; *r* = −0.506 (large), *p* = 0.019; respectively). Per cent changes in CT and SL correlated significantly with the 5000-m race time (Figure 4).

## 4. Discussion

In this study, we demonstrated a significant correlation between step characteristics (higher SF and SL normalized to BH) and race performance in endurance runners during a 5000-m race. Increases in SF and SL were found to increase running speeds [8,10]. Thus, our findings are corroborated by findings of previous studies, which show that a higher SF and SL are advantageous to race performance. Furthermore, we found significant correlations between longer SL and higher race performance during the second half of the 5000-m race, while they did not correlate during the first half of the 5000-m race in endurance runners. Additionally, smaller decreases in SL from the first half to the second half of the 5000-m race correlated with higher race performance in endurance runners. To the best of our knowledge, no other studies have examined the relationship between changes in SL and running performance. Therefore, the present study is the first to determine that smaller decreases in SL, in addition to higher average SF, are advantageous for achieving higher running performance in endurance runners.

In the second half of the 5000-m race, higher SL was related to better race time. Moreover, a smaller decrease in SL from the first half to the second half of the 5000-m race was also related to better race performance. Maintaining muscle force production against the onset of fatigue during maximal voluntary endurance running is an important factor for successful race performance [18,22]. Maintaining SL, rather than SF, would require a higher level of force production from the limb muscles [20,31]. Moreover, Hanley et al. [32] reported that runners maintained the same joint angles and foot positioning at initial contact throughout the 5000-m race while SL and SF decreased. This previous result indicated that local muscular endurance, not kinematic change, would have an important role to maintain SL and SF. Considering all the data, runners who are able to minimize a fatigue-induced decrease in SL perform better in races. Presumably, these runners can maintain muscle force production despite the onset of fatigue.

It is difficult to control for running speed in a real-race situation; therefore, the relationship between changes in step characteristics and fatigue onset time remains unclear. In a real-race situation, runners may act to save energy for the final sprint, thereby rendering it difficult to clarify whether the changes in running speed and SL are attributed to the onset of fatigue or to the strategical race plan. Further studies are warranted to confirm the relationship between step characteristics and running-induced fatigue when running at a constant race pace.

Like SL, average CT in the second half of the 5000-m race correlated with better race performance, although average CT in the overall and the first half of the 5000-m race did not correlate with race performance. Moreover, smaller increases in CT from the first half to the second half also correlated with a better 5000-m race time. Several studies have reported an increase in CT due to fatigue, particularly neuromuscular fatigue [18,33]. Shorter CT would be achieved throughout muscle pre-activation before ground contact and immediate transition between the eccentric and concentric phases during ground contact by the stretch-shortening cycle [33]. Nummela et al. [18] reported that CT increased with a decrease in the pre-activities of the limb muscles (i.e., the gastrocnemius and vastus medialis), which decreased due to running-induced fatigue throughout the 5000-m time trial. Additionally, they determined the relationship between change in CT and change in pre-activities of limb muscles during the 5000-m time trial. Therefore, there is supporting evidence that runners experiencing less fatigue may maintain their running speed by mitigating an increase in CT.

The highest recorded running speeds were noted in the final lap (i.e., final sprint of the race) similar to that noted in other studies [34,35]. During this lap, both higher and average SF and SL normalized to BH correlated with better race performance. Therefore, a training program designed to increase SF and SL would be important for better performance in the final sprint as well as the overall race.

Based on the findings of our studies, we concluded that smaller changes in SL and CT are advantageous for achieving better race performance because they are associated with lower running-induced fatigue. Maintaining a consistent running pace is an effective pacing strategy for maximizing performance in endurance races, and an over-running pace in the early stage of the race has a risk of developing excess fatigue and decreasing the running pace in the late stage of the race [35,36,37]. Taken together, maintaining a consistent running pace, which can minimize changes in SL and CT would be an important race strategy to achieve superior race performance in endurance races. Therefore, monitoring the step characteristics during the race may be helpful for evaluating and setting the race strategy in endurance runners.

Previous intervention studies have reported that resistance training, including plyometric training, can improve running performance by increasing SL [38,39]. Gómez-Molina et al. [39] reported that eight weeks of plyometric training increased SL and flight time and reduced SF during submaximal running in recreational runners. Esteve-Lanao et al. [38] reported that eight weeks of specified strength training could minimize the effects of running-induced fatigue on SL in well-trained middle-distance runners during the later stages of an endurance run. Thus, resistance training would be useful for increasing and maintaining SL over longer distances. Additionally, Quinn et al. [40] reported that high SF running training improved running economy with increasing SF. Therefore, information from the present and previous studies may be useful in facilitating the outlining of training programs in endurance runners by evaluating step characteristics during the race.

This study has several limitations. Firstly, only male Japanese endurance runners were recruited; therefore, the findings cannot be generalized to include runners of other races or other genders. A previous study has reported differences in step characteristics between male and female endurance runners [29]. Previous studies have also reported race-related differences in step characteristics based on morphological variations [13,14]. For the present study findings to be more universally applicable, further studies are needed with greater participant diversity. Secondly, the 5000-m race times correspond with the sub-elite to elite level, and three participants were considered elite runners (race times of less than 870 s). Further validation of the current statistical approach may arise from studies conducted using participants all running at the same optimal level (i.e., the elite level only). Finally, the present study followed a cross-sectional design, and the causal relationship between step characteristics and race performance was not investigated. There is a need for more longitudinal studies to fully evaluate this relationship and its impact on training for performance in endurance runners.

## 5. Conclusions

The present study found that a higher SF and SL normalized to BH correlated with better race performance in endurance runners. Furthermore, we were able to demonstrate that during a given race, smaller changes in SL correlated with better overall race performance. These findings suggest that consistency in SL is critical for achieving optimal running performance, and training for SL enhancement should be considered when developing programs for endurance runners.

## Figures and Tables

**Table 1:** Mean values of step characteristics in the 5000-m race.

Step Characteristics	Mean ± SD	Range
Average of all laps		
CT, s	0.170 ± 0.011	0.152–0.197
FT, s	0.145 ± 0.014	0.112–0.179
SF, Hz	3.16 ± 0.11	2.99–3.39
SL, m	1.79 ± 0.06	1.69–1.89
SL normalized to BH	1.05 ± 0.04	0.96–1.14
Average of the first half		
CT, s	0.168 ± 0.010	0.150–0.193
FT, s	0.145 ± 0.014	0.114–0.175
SF, Hz	3.19 ± 0.11	3.04–3.42
SL, m	1.80 ± 0.06	1.68–1.92
SL normalized to BH	1.06 ± 0.05	0.96–1.14
Average of the second half		
CT, s	0.174 ± 0.012	0.154–0.202
FT, s	0.146 ± 0.015	0.109–0.183
SF, Hz	3.13 ± 0.12	2.92–3.36
SL, m	1.77 ± 0.06	1.66–1.86
SL normalized to BH	1.04 ± 0.05	0.95–1.13
Final lap		
CT, s	0.158 ± 0.017	0.133–0.198
FT, s	0.141 ± 0.018	0.100–0.164
SF, Hz	3.37 ± 0.20	3.04–3.81
SL, m	1.90 ± 0.10	1.69–2.11
SL normalized to BH	1.11 ± 0.06	0.97–1.21
Per cent change		
CT, %	3.47 ± 3.75	−3.76–10.65
FT, %	0.36 ± 4.00	−7.48–8.95
SF, %	−1.96 ± 1.63	−4.73–0.63
SL, %	−1.70 ± 2.13	−7.75–1.58

## Data Availability

The data presented in this study are available on reasonable request from the corresponding author.
